# Surface Basicity and Hydrophilic Character of Coal Ash-Derived Zeolite NaP1 Modified by Fatty Acids

**DOI:** 10.3390/molecules29040768

**Published:** 2024-02-07

**Authors:** Ana-Paola Beltrão-Nunes, Marçal Pires, René Roy, Abdelkrim Azzouz

**Affiliations:** 1 Nanoqam, Department of Chemistry, University of Quebec at Montreal, Montreal, QC H3C 3P8, Canada; anapaolabn@gmail.com (A.-P.B.-N.); roy.rene@uqam.ca (R.R.); 2 Graduation Program on Engineering and Technology of Materials, School of Technology, Pontifical Catholic University of Rio Grande do Sul, Porto Alegre 90619-900, Brazil; 3 Station Expérimentale des Procédés Pilotes en Environnement, École de Technologie Supérieure, Montreal, QC H3C 1K3, Canada

**Keywords:** zeolite NaP1, fatty acids, carbon dioxide, thermal programmed desorption, basicity

## Abstract

Zeolite NaP1 was found to display the highest affinity for CO_2_ in preliminary modifications of coal fly ash-derived zeolites (4A, Y, NaP1 and X) by four amines (1,3-diaminopropane, *N*,*N*,*N*′,*N*′-tetramethylethylenediamine, Tris(2-aminoethyl)amine and ethylenediamine). In the second step, different fatty acid loaded NaP1 samples were prepared using palmitic, oleic and lauric acids. CO_2_ and H_2_O thermal programmed desorption (TPD) revealed changes in intrinsic basicity and hydrophilic character, expressed in terms of CO_2_ and H_2_O retention capacity (CRC and WRC, respectively). Infrared spectroscopy (IR), N_2_ adsorption-desorption isotherms and scanning electron microscopy allowed for correlating these changes with the type of interactions between the incorporated species and the zeolite surface. The highest CRC values and the lowest CO_2_ desorption temperatures were registered for NaP1 with the optimum content in palmitic acid (PA) and were explained in terms of the shading effect of surface acidity by the rise of basic Na^+^-palmitate salt upon cation exchange. The amine/fatty acid combination was found to paradoxically mitigate this beneficial effect of PA incorporation. These results are of great interest because they demonstrate that fatty acid incorporation is an interesting strategy for reversible CO_2_ capture.

## 1. Introduction

Emissions of greenhouse gases (GHG), including volatile organic compounds (VOC), and more particularly, carbon dioxide (CO_2_) and methane (CH_4_), are major contributors to global warming. CH_4_, a nonpolar molecule, is the second-largest GHG after CO_2_ in terms of global warming potential (GWP), but it is even ca. 21–25 times more effective in adsorbing radiant energy than CO_2_ [1,2]. Another form of pollution is that related to acid rain, produced by the dissolution of gaseous nitrogen and sulfur oxides (NO_x_ and SO_x_).

An interesting avenue to explore resides in reusing solid wastes released by industries and mining to simultaneously address two environmental issues: (1) the capture of polluting gases and (2) the remediation of pollution caused by the very solid wastes [3]. Steel slag, red mud, biogas wastes and wood wastes have already been used as sources of adsorbing materials [4]. The conversion of coal fly ash (CFA) results in CFA-derived zeolites [5,6,7,8]. The latter are low-cost and value-added products, in spite of their lower effectiveness in CO_2_ capture compared to commercially available adsorbents [9,10]. In addition, their detrimental surface acidity and hydrophilic character pose challenges for the capture of acidic gases and VOC. Adequate surface basicity and hydrophobic character may be tailored for such purposes through suitable modifications, such as amine incorporation. Nonetheless, these modifications often lead to decreases in surface area and pore volume [11,12,13], more particularly when dealing with zeolite frameworks [14,15,16,17,18,19,20,21,22,23,24,25].

Up until now, amine-modified zeolites derived from CFA, and more particularly, NaP1, have attracted special interest. The special interest devoted herein to NaP1 arises not only from its intrinsic adsorptive properties, which have barely been investigated so far [26,27,28,29], but also from the beneficial incorporation of organic species [30,31]. CO_2_ chemically adsorbs on amines via the unavoidable formation of carbamate, which requires heating for the regeneration of amine-functionalized adsorbents [9,13,32,33]. Since the CO_2_:amine reaction is favored by high amine content [25,34], lower amine loadings should promote weak retention with an easier release of CO_2_ [35].

Here, achieving moderate surface basicity with optimal amine content still remains a challenge. Substituting amines with other chemical species is an original approach to mitigate surface basicity. Scarce attempts achieved thus far revealed that OH-enriched clay minerals allow the achievement of truly reversible CO_2_ capture but at the expense of CO_2_ retention capacity [13,36,37,38,39,40,41]. The incorporation of fatty acids (FAs) is another strategy, which can only be justified by their sources from animal fats (long chain) or from industrial wastes [42,43,44,45]. FA adsorption on zeolite surfaces should be favored by the presence of hydrophobic Si–O–Si rich islands, if any, and by acidic sites [43], which may also promote H-bridges with FA carboxylic groups (–COOH) [44]. The latter confers Bronsted acidity and a polar character to low molecular weight FA. The carboxyl polarity that governs the acidity strength and hydrophobicity of the FA-modified zeolite depends on the length of the organic chain [46]. This effect was herein studied with three fatty acids.

High CO_2_ retention capacity (CRC) requires high surface basicity, while high water retention capacity (WRC) imposes low hydrophobicity due to the Lewis acidity of the gas. These two features were measured for zeolite NaP1 after modification using thermal programmed desorption of CO_2_ and water [29], and they were compared to those of commercial zeolite Y and other CFA-derived zeolites (4A and X). The effect of the FA–amine combination was also examined by incorporating the three FA studied in one of the NaP1-amine samples displaying the highest CRC among those modified with the four different amines.

## 2. Results and Discussion

### 2.1. Effect of Zeolite Modification

The FTIR analysis of NaP1-DAP revealed an intensity increase for the common characteristic bands at 3700–3000 cm^−1^ (N–H bond stretching) and around 1625 cm^−1^ (N–H bond bending) as compared to NaP1 (Figure S2). This phenomenon is a common feature for all amines (Figure S3) and zeolites investigated herein (Figures S4 and S5). These two bands may also overlap contributions of the following: (1) an O–H stretching vibration of H-bonded and adsorbed water molecules and (2) H–O–H bending vibration, given that all amine-loaded zeolites instantly adsorb moisture during IR analysis or when handled/transferred outside the sealed storage enclosure. Evidence in this regard was provided by the mere detection of moisture by water-TPD measurements. The rise of the 2425 cm^−1^ and 1385 cm^−1^ bands provides clear evidence of a spontaneous capture of gas phase CO_2_ and the formation of carbonate anion (CO_3_^−^) even at ambient conditions [47]. The 2425 cm^−1^ band was already observed after exposure to air of NaP1 previously stored in a dry CO_2_-free enclosure for several days (Figure S6).

**Scheme 1:**
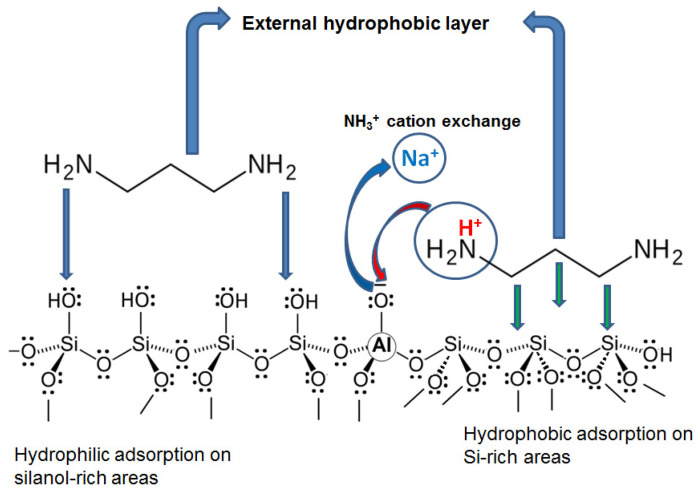
Reversed hydrophobicity and possible surface interactions on DAP loaded zeolites. Cation-exchange between Na^+^-zeolite and ammonium group of amine results in external surface exhibiting hydrophobic character with Na^+^-ammonium salt.

**Scheme 2:**
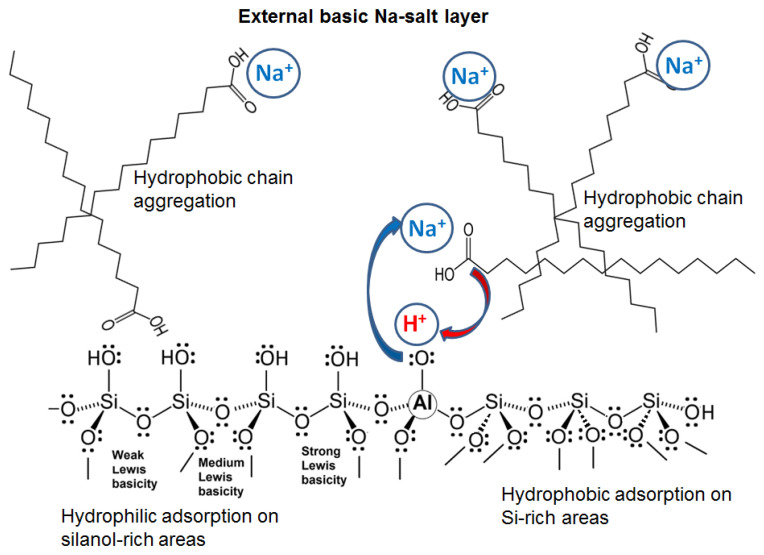
Reversed acidity of the surface and possible interactions on palmitic acid-loaded zeolites. Cation-exchange between Na^+^-zeolite and carboxylic group of palmitic acid results in external surface exhibiting amphoteric Na^+^-palmitate salt.

Here, the most plausible explanation resides in the following: (1) a DAP adsorption via hydrophobic interaction of the propyl chain that shades the hydrophobic character of the silica-rich areas on the zeolite surface (Scheme 1); (2) an additional contribution of the incorporated hydrophilic amino groups. At the alkaline pH of the zeolite impregnation mixture, hydrophobic amine adsorption should prevail at the expense of possible competitive interactions of the amino groups. The latter may involve the formation of H-bridges with terminal silanols (–Si–OH) and aluminols (–Al–OH), and/or chemosorption by cation exchange after amino group protonation.

Amine incorporation gave rise to new peaks, in addition to the original 1000 cm^−1^ band attributed to the Si–O stretching vibration of the Si–O–Si structure and 750 cm^−1^ peak due to Al–O–Al groups of zeolites [11,22,25,32,35,48,49,50,51,52,53]. These bands rather appeared as barely detectable shoulders at 1250–1020 cm^−1^ (C–N-bond stretching) and 910–665 cm^−1^ (N–H wagging). Unlike tertiary amines, their primary counterparts also showed two bands corresponding to asymmetrical and symmetrical N–H stretching modes and to an N–H bending vibration in the region 1650–1580 cm^−1^. These bands could not be detected for secondary amines.

FA incorporation in amine-modified NaP1 generated a strong and broad band in the region of 3600–3000 cm^−1^ that overlaps with those assigned to O–H bond stretching beyond 3000 cm^−1^ and C–H stretching of both alkyl and aromatic groups. Here, FA adsorption on zeolite surfaces is assumed to involve not only a similar interaction of the organic chain with hydrophobic Si–O–Si rich islands, if any, but also with the acidic sites [43] and H-bridge formation with FA carboxyls (–COOH) [44].

This agrees with the broadening of the O–H stretching band of carboxylic acids that may arise from the unavoidable interaction (dimerization) of carboxylic acids through H-bridges. The carbonyl bond stretching (C=O) of FAs appears as an intense band at 1760–1690 cm^−1^. The C–O bond stretching was observed in the region 1320–1210 cm^−1^, and the O–H bending at 1440–1395 cm^−1^ and 950–910 cm^−1^. It is worth mentioning that the 1440–1395 band might not be distinguished from that of C–H bending [54,55,56,57,58,59,60,61]. Here also, the rise of these new IR bands is a precise indictor of FA incorporation.

Loading NaP1 with a DAP and PA combination resulted in NaP1-DAP-PA samples displaying a complex FTIR spectrum with all characteristic bands for amines at 1650–1580 cm^−1^ and for FAs at 3700–3000 cm^−1^ and 1780–1710 cm^−1^ (Figure S3). Interestingly, the intensities of these bands are slightly higher for NaP1-DAP-1-PA-10 as compared to NaP1-DAP-10 (Figure S1) but lower than those of NaP1-DAP-1. This must be due to a contribution of the following factors: (1) an O–H stretching vibration of H-bonded and adsorbed water molecules and (2) H–O–H bending vibration. In addition, the hydrophilicity enhancement upon amine incorporation agrees with previous statements and is supported by the excessive broadness of the 3700–3000 cm^−1^ band.

### 2.2. Textural Property Changes upon Zeolite Modification

The incorporation of palmitic acid (PA) produced no detectable changes in the sharp and well-resolved XRD peaks of zeolite NaP1, and there was no crystallinity loss (Figure S7). The lines appearing at 2θ = 16.2, 26 and 41 are related to the mullite phase and that observed at 2θ = 28 to the quartz phase [29]. This appears to be a common feature after the incorporation of all organic moieties. However, the incorporation of amines and FAs was found to reduce the specific surface area (SSA) from 38 m^2^.g^−1^ for the starting NaP1 [29] down to 28 m^2^.g^−1^ after 1% PA loading. This was accompanied by a dramatic decay in pore volume from 0.200 cm^3^.g^−1^, which agrees with the literature [2], down to 0.023–0.096 cm^3^.g^−1^, the most pronounced being for high 10–30% FA loading (Table 3). This suggests that the incorporation of organic species occurs mainly on the external surface of the zeolite crystallites, inducing pore entry obstruction. This appears to affect the hydrophilic character, as reflected by the pronounced WRC decrease from 1.72 to 0.01–0.42 µmol.g^−1^.

Increasing the PA content induced more pronounced decreases in SSA and pore volume depletion and an increase in pore diameter. Indeed, raising PA loading to 1, 5 and 10% induced no change in the chemical composition of the starting NaP1 (Table S1) but resulted in an increase in the average pore diameter from 2.0 up to 85, 340 and 513 Å, respectively. Incorporating 10% OA and LA resulted in macropores (7477 Å for 10% OA) and a very low SSA, even leading to total porosity suppression. This must be due to the increase in interparticle void space as organo-zeolite aggregates into larger clusters exceeding a 20–30 µm particle size (Figure S8). Therefore, maintaining low FA contents appears to be an essential requirement to avoid a loss in textural properties, in agreement with previous works [34].

The fact that the incorporation of 1% DAP induced a more pronounced SSA decay, decreasing to 2 m^2^.g^−1^ with a pore diameter of up to 1197 Å as compared to 1% EDA (20 m^2^.g^−1^ and 171 Å, respectively), suggests strong effects of the molecular structure and polarity of the organic species on their interaction and dispersion on the zeolite surface. The slight revival of the SSA (4.5–6.0 m^2^.g^−1^) and pore volume (0.042–0.44 cm^3^.g^−1^) for higher PA loading of 20–30% in NaP1 must be due to the rise of competitive organophilic and electrostatic attraction forces between the FA molecules at the expense of FA: zeolite interaction. This remains to be elucidated through deeper insights into this phenomenon.

### 2.3. Basicity Improvement upon Amine Insertion

As a common feature, TPD profiles revealed the appearance of a single desorption peak after the incorporation of all amines investigated, regardless to the zeolite (Figure S9). This accounts for a uniformization of the surface basicity, most likely due to a shading effect of amine basicity on the distribution of basicity strength of the starting unmodified zeolites [29]. However, this uniform basicity was found to vary in intensity (peak area) and strength (desorption temperature) according to the zeolite due to the specific formation of carbamates [25,34] through CO_2_ reaction with each amine [17].

**Figure 1:**
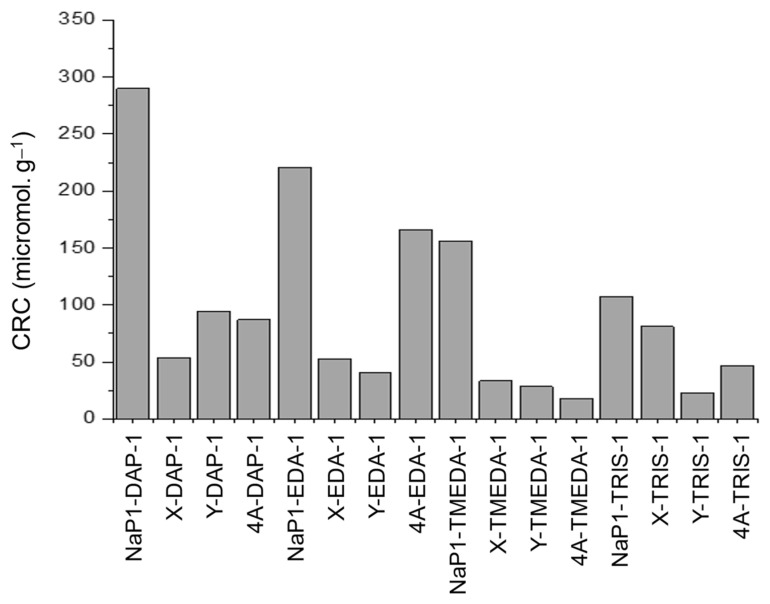
CRC for zeolites 4A, X, NaP1 and Y loaded with 1% of different amines.

On the one hand, NaP1/DAP, NaP1-EDA, 4A/EDA and NaP1/TMEDA showed the highest peak intensity, and thereby the highest basicity in decreasing order. On the other hand, NaP1-EDA-1 and NaP1/TMEDA showed a shifted STD peak under the same carrier gas stream, indicating a stronger retention of CO_2_ as compared to almost all modified zeolites. Additionally, NaP1/TRIS showed a lower peak intensity at a lower desorption temperature than NaP1/EDA, indicating that triamines do not necessarily exhibit stronger basicity than diamines. This is in agreement with the lower CRC of NaP1-TRIS (107.5 µmol.g^−1^) compared to NaP1/EDA (220.8 µmol.g^−1^) (Figure 1 and Table S2).

This finding confirms, once again, the effects of the structures of amines and zeolites, as well as their interactions, on their specific surface area and pore volume [9,11,14,15,16,19,21,22,24,33,62,63]. The lower CRC of 1% amine-loaded zeolites 4A, X and Y, more particularly with TRIS and TMEDA as compared to their NaP1-based counterparts, can be explained by the higher alkalinity of the starting NaP1.

This alkalinity is rather induced by its higher Ca and Mg content (1.14–116 wt.% and 0.57–0.58 wt.%, respectively) [29], unlike that of unwashed NaP1, which arises from residual amounts of coal fly ashes and basic oxides, such as CaO and MgO, silica (SiO_2_), alumina (Al_2_O_3_), lime (CaO) and iron oxide (Fe_2_O_3_) with traces of unburned coal [10,64,65,66,67,68,69]. In addition, the pore volume of NaP1 (0.2 cm^3^.g^−1^) is similar to that of zeolite 4A and by far lower than those of faujasites X (2.0 cm^3^.g^−1^) and Y (3.3 cm^3^.g^−1^). This accounts for the lower framework density, which induces higher basicity, given that acidity strength decreases with increasing density [70]. This is why a special interest was devoted to zeolite NaP1.

**Figure 2:**
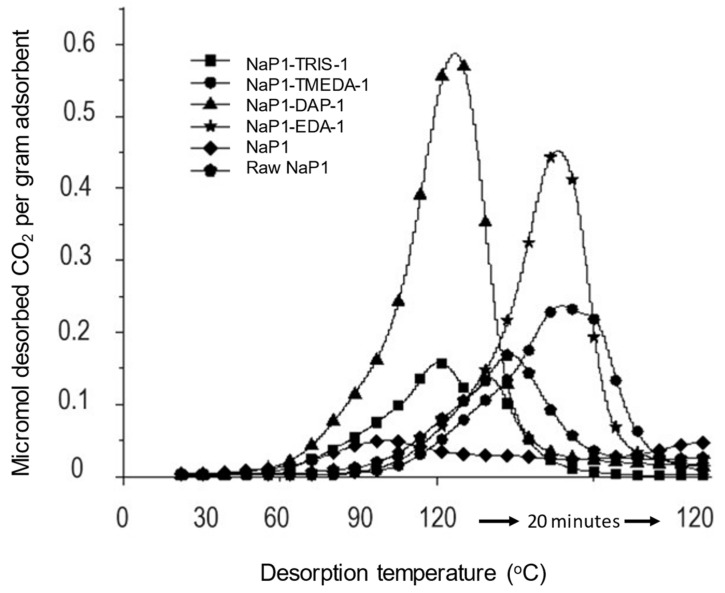
CO_2_ TPD profile of amine-loaded zeolites between 20 and 120 °C and stepwise thermal desorption (STD) at constant temperature of 120 °C for 20 min. These patterns correspond to combined TPD-STD diagrams of NaP1 loaded with 1% of different amines. The STD step was performed to avoid measurement accuracy alteration due possible thermal degradation of amines.

NaP1 displayed a higher affinity towards CO_2_ after diamine incorporation (EDA and DAP) as compared to polyamines (TRIS and TMEDA) (Figure 2). The latter are assumed to involve excessive interaction with the zeolite, inducing a pronounced surface coating that shades the intrinsic surface basicity of NaP1, in agreement with previous statements. As expected, amine incorporation resulted in a visible shift of the main CO_2_ desorption peak of NaP1 from approximately 100 °C to 120 °C under STD mode due to the rise of stronger basicity. The less pronounced shift registered with DAP and TRIS incorporation makes the corresponding NaP1 samples be regarded as the most suitable for a reversible and non-stoichiometric CO_2_ capture [36,37,38,39,40,41,71,72].

### 2.4. Effect of Fatty Acid Incorporation

**Figure 3:**
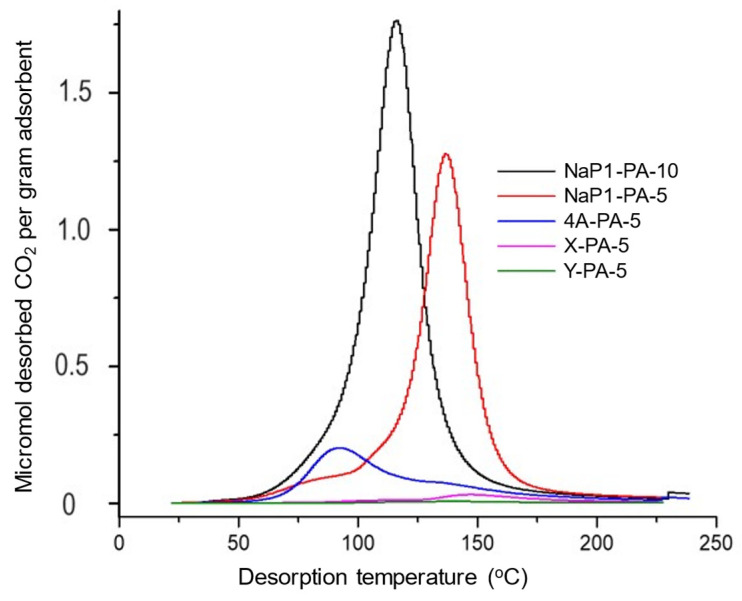
CO_2_ TPD profile between 20 and 120 °C of zeolite-PA-5 samples and stepwise thermal desorption (STD) at constant temperature of 120 °C for 20 min. These patterns correspond to combined TPD-STD diagrams of different zeolite loaded with 5% of palmitic acid. The zeolite-FA materials were saturated with dry CO_2_ for 24 h in a static mode and then purged of the excess of CO_2_ at RT under a dry N_2_ stream. The perfectly symmetrical TPD peaks up to 200 °C support the higher stability of fatty acids as compared to amines, as assessed through preliminary thermogravimetric measurements.

**Figure 4:**
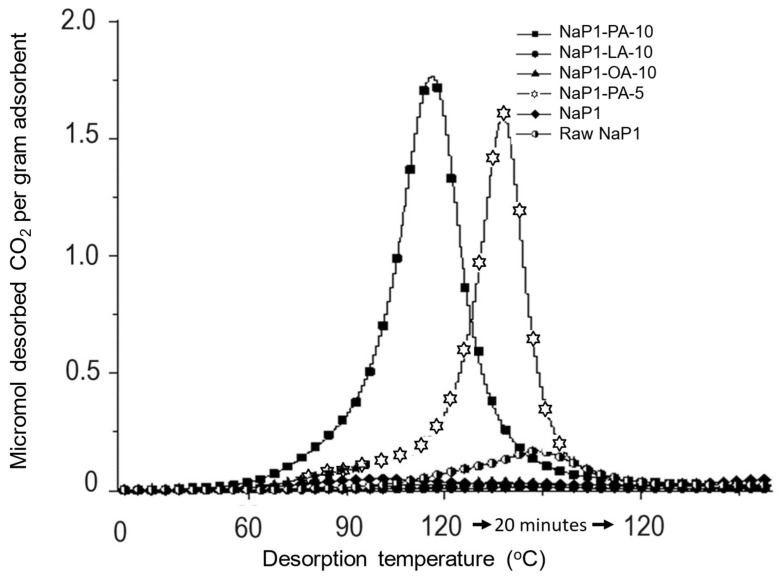
CO_2_ TPD profile between 20 and 120 °C of NaP1-PA-10 and stepwise thermal desorption (STD) at a constant temperature of 120 °C for 20 min. These patterns correspond to the combined TPD–STD diagrams of zeolite NaP1 loaded with 10% of different fatty acids. The zeolite-FA materials were saturated with dry CO_2_ for 24 h in a static mode and then purged of the excess of CO_2_ at RT under a dry N_2_ stream. The additional STD step at 120 °C was only performed for the comparison of the individual effects of the different FAs and contents, around a similar maximum desorption temperature as for amines.

The incorporation of 5% of palmitic acid in NaP1 induced a significant intensity increase of the desorption peak of CO_2_ (Figure 3). This effect was barely detectable for the other zeolites, thus confirming the special interest devoted to NaP1. The effect of a higher FA content of 10% was illustrated by NaP1/PA-10, and to a lesser extent, NaP1/PA-5, both of which showed by far more intense CO_2_ desorption peaks as compared to the barely detectable peaks of NaP1-OA-10 and NaP1-OA-10 (Figure 4). The highest CRC value obtained for NaP1-PA-10 (662.49 µmol.g^−1^) contrasts with its low SSA (3 m^2^.g^−1^) and porosity (0.033 cm^3^.g^−1^) (Table 3).

This suggests a preponderant multilayer adsorption of CO_2_ via non-stoichiometric condensation involving weak and purely physical interactions with outermost functional groups [73]. This is assumed to take place on a compacted structure resulting from an aggregation of organo-zeolite grains into bulkier clusters with large intergranular pores, in agreement with the high pore size (513 Å). Here, the excessive formation of H-bridges between the carboxyl group of FA molecules and the zeolite Al–OH–Si groups must be involved [44,74]. In such a compacted structure, increasing the FA content may also enhance hydrophobic interactions, and CO_2_ internal diffusion, if any, should have only a minor contribution [28].

### 2.5. Effect of Fatty Acid Content

The CRC improvement, from 589.89 µmol.g^−1^ up to 662.49 µmol.g^−1^, observed with increasing PA content from 1% to 10% (Table 3), can be explained by a progressive surface coverage that shades the exchangeable sites and BrØnsted acidity (Scheme 2). Here, the amount of adsorbed FA molecules is supposed to increase with higher Al content [29], i.e., with a lower Si/Al ratio.

Increasing PA contents by 20 and 30% induced a marked CRC decrease down to 67.28–85.42 µmol.g^−1^, respectively (Table 3). This suggests a weaker shading effect on surface acidity, which revives BrØnsted acidity and affects the affinity towards CO_2_. A possible explanation resides in the rise of competitive organophilic FA:FA chain interaction and FA molecule aggregation into compact organic clusters at the expense of FA adsorption and surface coverage. This effect of FA content must vary according to the FA structure and the Si/Al ratio of the zeolite. Increasing the FA content from 1% to 10% resulted in only a slight CRC improvement, with oleic acid increasing from 29.91 µmol.g^−1^ up to 41.78 µmol.g^−1^ and lauric acid decreasing from 22.13 µmol.g^−1^ down to 18.68 µmol.g^−1^.

The fact that the average CRC value paradoxically decreased in the following sequence PA > OA > LA, i.e., with increasing pKa or increased basicity, suggests that the carboxyl groups are not involved in CO_2_ adsorption but rather in interactions with the Si–O–Al sites via Na^+^ cation exchange (Scheme 2). The formation of basic Na-salts over the protonated surface can explain somehow the shift of the main CO_2_ desorption peak of NaP1 from 100 °C to 120 °C, as confirmed by peak deconvolution of the CO_2_ TPD patterns of FA-modified NaP1-10 (Figure S10) and of the starting NaP1 [29].

### 2.6. Effect of Amine–FA Combination

Deeper insights into PA incorporation in amine modified NaP1 revealed a general tendency of CRC decay (Table 1). This CRC decrease was more pronounced for NaP1-TMEDA-1-PA (from 156.4 to 29.70–3.69 µmol.g^−1^) as compared to those in the other samples and appears to be enhanced by increasing the PA content from 1 to 10%. The second more pronounced CRC decrease occurred for NaP1-EDA-1-PA (from 220.8 to 206.78–60.22 µmol.g^−1^), i.e., also involving an ethylenediamine as a common feature. This may be explained by the occurrence of a combination of hydrophilic-hydrophobic interactions between both incorporated species and simultaneously with the zeolite surface. This requires a deeper investigation into this aspect.

### 2.7. Hydrophilic Character

As previously stated, the mere incorporation of organic species induced a marked decay in hydrophilic character. The role of FA pKa is unclear and should be investigated in correlation with the hydrophilic-hydrophobic character of the zeolite surface. Deeper insights, gained through water-TPD measurements in the water retention capacity (WRC), showed no clear correlation with the CRC values. This is due to the different structures and content of the incorporated organic species in the different host-zeolites. which determine multiple interactions that still remain to be investigated. The hydrophilic character, expressed in terms of water retention capacity (WRC), was found to increase in the reverse sequence (LA > OA >> PA), i.e., with increasing FA basicity, for the same FA content range (1–10%). This suggests that moisture does not contribute, or at least contributes weakly to CO_2_ capture, unlike on OH-rich organoclays [36,37,38,39,40,41,71].

**Figure 5:**
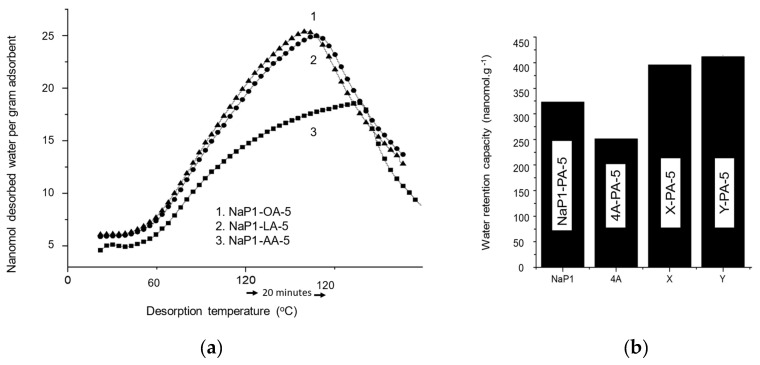
Water-TPD patterns of NaP1-FA-5 samples (**a**) and comparison with zeolite-PA-5 samples (**b**). All samples were saturated with dry CO_2_ for 24 h in a static mode, and the excess of non-adsorbed CO_2_ was then purged at RT under a dry N_2_ stream. These TPD patterns (**a**) are combined with stepwise thermal desorption (STD) at a constant temperature of 120 °C for 20 min. The additional STD step was only performed for a comparison of the individual effects of the different FAs and contents around a similar maximum desorption temperature as for amines. NaP1-FA-5: NaP1 loaded with 5% of different fatty acids. Zeolite-PA-5: different zeolites loaded with a similar 5% content in palmitic acid.

NaP1-PA-5 showed the lowest intensity of the water desorption peak, with a shift toward a higher temperature as compared to NaP1-OA-5 and NaP1-LA-5 (Figure 5a). This must be due to its longer saturated organic chain, which is assumed to induce stronger hydrophobic interactions with organophilic Si–O–Si groups of NaP1, as already reported in the literature [75]. Therefore, water adsorption should be restricted to the uncovered hydrophilic exchangeable sites (Scheme 2).

Similar FA contents improve differently the hydrophilic character according to the zeolite structure. NaP1-PA-5 showed a lower WRC value (320 nanomol.g^−1^) as compared to X-PA-5 (400 nanomol.g^−1^) and Y-PA-5 (420 nanomol.g^−1^) (Figure 5b). This can be explained by the contribution of the higher hydrophilic character of the bare zeolite [29]. However, NaP1-PA-5 displayed a higher CRC than that of 4A-PA-5 (250 nanomol.g^−1^), presumably due to the lower pore size and pore volume of zeolite 4A.

**Figure 6:**
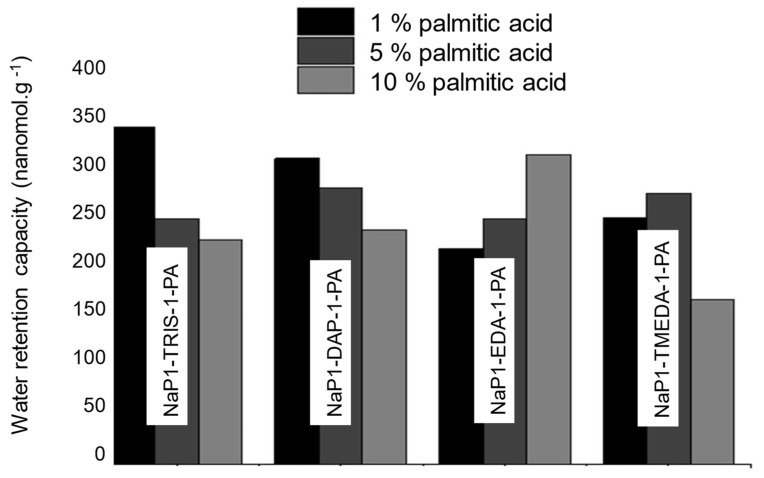
Effect of increasing palmitic acid content on the water retention capacity of different zeolites loaded with 1% amine.

When combined with different amines, this tendency was altered, as evidenced by the highest WRC values registered for NaP1-TRIS-1-PA-1 (330 nanomol.g^−1^) and paradoxically for NaP1-EDA-1-PA-10 (300 nanomol.g^−1^) (Figure 6). A possible explanation resides in the rise of additional FA: amine interactions at the expense of those occurring between the incorporate species and the zeolite surface. Investigations have to be pursued in this direction because the magnitude of these interactions is expected to play a key-role in tailoring optimum basicity and hydrophobic character for improved CO_2_ capture. Except for a few data related to the key-role of FA loading in zeolite-catalyzed processes [76] and the hydrophobic-hydrophilic and acid-base interactions [77], there exists a glaring lack of specific studies on FA adsorption on zeolites.

## 3. Materials and Methods

### 3.1. Zeolite Synthesis and Modification

Zeolites were synthesized through a two-step hydrothermal process using coal fly ash (CFA) according a procedure fully described elsewhere [29]. CFA was obtained from coal combustion at the thermal power plant President Medici (UTPM) in Candiota City, Rio Grande do Sul, Brazil [6]. Three types of zeolites (NaP1, 4A and X) were synthetized starting from a 99% dry CFA sample characterized by a silica/alumina weight ratio of 1.99 and some major impurities, such as Fe_2_O_3_ (4.80%), K_2_O (2.72), CaO (1.44) and TiO_2_ (1.40) [29]. Zeolites 4A and X showed higher purity as compared to raw NaP1, which was found to contain quartz and mullite, among other impurities. Further washing of NaP1 with deionized water until pH 7 resulted in a sample denoted as NaP1. The characterization of all synthesized zeolites and a commercial Y-faujasite (IQE, Spain) used for comparison has already been reported [29]. In preliminary attempts, these zeolites were loaded with various amines (Table 2).

For this purpose, 10 mg of each amine, including 1,3-diaminopropane (DAP, 99% purity from Aldrich, St. Louis, MO, USA), ethylenediamine (EDA, p.a. from Acros Organics, Geel, Belgium), *N*,*N*,*N*′,*N*′-tetramethylethylenediamine (TMEDA, Bio-Rad Laboratories, Hercules, CA, USA) and Tris(2-aminoethyl)amine (TRIS, 96% purity from Aldrich), were added to 1 g of zeolite in 10 mL of anhydrous ethanol. The resulting mixture was continuously and vigorously stirred for 12 h at room temperature (RT), then dried under vacuum at 60 °C for ca. 45 min, according to previous works [25,34,51,53]. CO_2_ capture tests on all modified zeolites allowed for the selection of NaP1 containing 1% DAP, denoted as NaP1-DAP-1, as being the most efficient adsorbent for CO_2_ (Table 3).

The latter and the bare NaP1 sample were loaded with different fatty acids to achieve deeper insights into the effect of the amine–FA combination and the FA incorporation. Palmitic acid (PA) (ACS, 98 Vol.%), a saturated medium strength FA with a 16-carbon chain, and slightly less acidic counterparts, such as oleic acid (OA) and lauric acid (LA), were employed for this purpose.

Thus, 1 g of NaP1 or NaP1-DAP-1 was dispersed in 10 mL of 1:1 vol. water-anhydrous ethanol mixtures containing various amounts of FA, then stirred for 24h at RT, and further dried under vacuum and heated at 70 °C for ca. 1h, resulting in dry NaP1-FA-X and NaP1-DAP-1-FA-X samples with X = 1, 5, 10, 20 and 30 Wt.% in FA content. Reportedly, drying at temperatures of up to 75 °C enhances oleic acid adsorption onto zeolite 13X. All zeolites and their modified counterparts were then stored in a dry CO_2_-free enclosure prior to further characterization.

### 3.2. Characterization

All synthesized zeolites, including the starting CFA and their modified counterparts, were characterized through X-Ray diffraction (XRD) (Siemens D5000 instrument, Munich, Germany, Co-Kalpha at 1.7890 Å) and X-ray fluorescence (XRF) (Brücker AXS S4 Pioneer instrument, Billerica, MA, USA). The particle morphology and chemical composition were evaluated by a scanning electron microscope (SEM, model XL 30 Philips, Amsterdam, The Netherlands) coupled to an energy dispersion X-ray fluorescence AX system (EDS). Nitrogen adsorption–desorption isotherms were used to assess the specific surface area (SSA) and porosity of bare and modified NaP1 samples, previously stored in a dry CO_2_-free enclosure. This was achieved by means of a Quantachrome instrument and a Quantachrome Autosorb gas sorption device (Boynton Beach, FL, USA). The samples were subjected to outgassing at 120 °C under vacuum for 12 h and analyzed using the Brunauer, Emmett and Teller (BET) and Barrett, Joyner and Halenda (BJH) models, respectively. About 0.4 g of each sample was degassed at 130 °C during 24 h before measuring the BET surface area, porous volume and porous diameter from the amount of liquid nitrogen adsorbed. Low temperature pre-treatment was chosen to prevent losses in amines and or fatty acids under high vacuum treatment. The Fourier transform infrared spectroscopy (FT-IR) analysis was performed in the range 400–6000 cm^−1^ using Model IR 550, Magna Nicolet equipment (SpectraLab Scientific Inc., Markham, ON, Canada).

### 3.3. Thermal Programmed Desorption (TPD) Measurements

The effects of amine insertion on surface basicity and hydrophilic character were comparatively investigated in terms of capacity of retention of CO_2_ (CRC) and water (WRC), respectively. The latter were assessed by thermal programmed desorption (TPD) using CO_2_ under a nitrogen stream (dry and O_2_-free N60 pure grade gases), as fully described elsewhere [29,71,72,78,79]. Both CRC and WRC were defined as the areas determined by the TPD patterns within the temperature range of thermal stability (20–160 °C), as previously provided by ample literature for amines and by preliminary TGA measurements for the three FAs investigated herein (Figure S1). This temperature range was restricted to 20–120 °C to achieve a viable CO_2_ capture, considering the thermal stability of the incorporated FAs. As a general tendency, supported fatty acids are more thermally stable than free molecules [80,81,82,83]. In addition, saturated and short chain fatty acid are the most stable below 300 °C, resulting in a decrease in thermal stability in the following sequence: lauric acid ˃ palmitic acid ˃ oleic acid [84,85,86,87]. Similar TPD patterns were achieved after repeated desorption–wet CO_2_ re-saturation cycles, providing clear evidence of the thermal stability of the investigated samples within the range of 20–120 °C, with a consecutive stepwise thermal desorption (STD) at a constant temperature of 120 °C for 20 min.

Thus, 40–50 mg samples with a 0.1–0.2 mm particle size were introduced in the form of a 2–3 cm zeolite bed into the cylindrical glass micro-reactor (2 mm internal diameter) of an oven connected to a Li-840A dual CO_2_/H_2_O gas analyzer (Li-COR Bioscience Inc., Lincoln, NE, USA). To achieve adsorption–desorption equilibrium, dry CO_2_ was injected until sample saturation for at least 30 min and a maximum time of 24h in a static mode, without a carrier gas stream. The excess CO_2_ of the probe gas was purged at RT with a dry 5 mL.min^−1^ N_2_ stream. First, TPD profiles were simultaneously performed for the released CO_2_ and moisture from both fresh and non-dehydrated samples under a 5 °C.min^−1^ heating rate, for 20 min (TPD1). The second and third TPD patterns (TPD2 and TPD3) were recorded on the cooled and non-rehydrated samples after re-saturation with dry CO_2_ under similar conditions.

## 4. Conclusions

The results obtained herein allow us to conclude that the incorporation of amines and fatty acids in CFA-derived zeolites induces marked changes in their absorptive properties. NaP1 loading with palmitic acid improved the affinity towards CO_2_ and reduced the gas retention strength, and thereby the energy consumption for adsorbent regeneration, but excessive loading beyond 10% turned out to be detrimental. The affinity for CO_2_ decreases with an increasing pKa of the fatty acid. The zeolite seems to behave as a solid acidic solution that imposes a base-like behavior to the adsorbed FA. In other words, carboxylate groups will act as a stronger conjugated base than the parent FA. These results cannot be explained by the marked loss in textural properties upon FA incorporation, but rather by the role of pKa in combination with a complex hydrophilic-hydrophobic interaction between the zeolite surface and each of the incorporated species. Zeolite modification with amines and/or fatty acids paradoxically affects the affinity towards CO_2_. The combination of amines and fatty acids is believed to induce a detrimental interaction between both amines and fatty acids on the zeolite surface. Palmitic acid incorporation in coal fly ash-derived zeolites is a prudent approach for simultaneously addressing two pollution issues caused by CO_2_ and coal fly ash release in the environment.

## Figures and Tables

**Table 1:** Effect of PA content on the CO_2_ and water retention capacity of 1% amine-loaded NaP1.

Amines (1%)	Amine Content (%)	Palmitic Acid (%)	CRC ^a^ (µmol.g^−1^)
None ^b^	0	0	296.10 ^c^
			60 ^d^
1,3-diaminopropane (DAP)	0	1	589.89
		5	483.10
		10	662.49
		20	67.28
		30	85.42
	1	0%	289.9
		1%	103.24
		5%	131.48
		10%	144.24
Ethylenediamine (EDA)	1	0%	220.8
		1%	206.78
		5%	91.58
		10%	60.22
*N*,*N*,*N*′,*N*′-tetramethylethylenediamine (TMEDA)	1	0%	156.4
		1%	29.70
		5%	22.78
		10%	3.69
Tris(2-aminoethyl)amine (TRIS)	1	0%	107.5
		1%	24.87
		5%	100.14
		10%	65.72

^a^ CRC: CO_2_ retention capacity (micromoles of desorbed CO_2_ per gram adsorbent). ^b^ NaP1: NaP1 washed. ^c^ CRC measured by TPD performed at a 5 °C.min^−1^ heating rate from 20 °C to 450 °C under a 5 mL min^−1^ nitrogen stream; ^d^ CRC measured by similar TPD but from 20 °C to 120 °C, followed by a stepwise thermal desorption (STD) at constant a temperature of 120 °C for 20 min.

**Table 2:** Structure of the amines and fatty acids incorporated in zeolites.

Amines	Symbol	pKa	Fatty Acids	Symbol	pKa
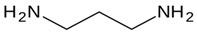 1,3-Diaminopropane	DAP	10.17	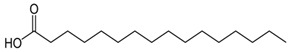 Palmitic acid C_16_H_32_O_2_	PA	4.95
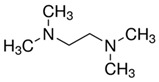 *N*,*N*,*N*′,*N*′-Tetramethylethylenediamine	TMEDA	10.40 and 8.26	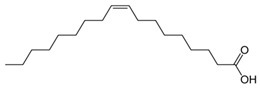 Oleic acid C_18_H_34_O_2_	OA	5.02
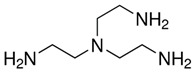 Tris(2-aminoethyl)amine	TRIS	9.42	Lauric acid C_12_H_24_O_2_ 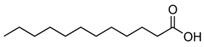	LA	5.30
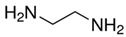 Ethylenediamine	EDA	10.0			

**Table 3:** Some features of zeolite NaP1 and its modified counterparts.

Incorporated Organic Species	Content (wt.%)	Samples ^a^	BET SSA ^b^ (m^2^.g^−1^)	Pore Features		CRC ^c^ (µmol.g^−1^)	WRC ^d^ (µmol.g^−1^)
				Size (Å)	Volume (cm^3^.g^−1^)		
None	0	Raw NaP1 ^e^	38	2.0	0.200	399.2	9.38 [29]
None	0	NaP1 ^e^	-	-	-	296.1 ^f^	1.72 [29]
						60 ^g^	
Palmitic acid (PA)	1	NaP1-PA-1	28	85.1	0.059	589.89	0.01
	5	NaP1-PA-5	11	340	0.096	483.10	0.32
	10	NaP1-PA-10	3	513	0.033	662.49	0.32
	20	NaP1-PA-20	6	291	0.044	67.28	0.25
	30	NaP1-PA-30	4.5	379	0.042	85.42	0.35
Lauric acid	1	NaP1-LA-1	-	-	-	22.13	0.40
	5	NaP1-LA-5	-	-	-	17.08	0.39
	10	NaP1-LA-10	4–5	281	0.031	18.68	0.41
Oleic acid	1	NaP1-OA-1	-	-	-	29.91	0.42
	5	NaP1-OA-5	-	-	-	39.22	0.40
	10	NaP1-OA-10	~0	7477	0.023	41.78	0.38
Diaminopropane	1	NaP1-DAP-1	2	1197	0.061	289.9	-
Ethylenediamine	1	NaP1-EDA-1	20	171	0.084	220.8	-
TMEDA	1	NaP1-TMEDA-1	-	-	-	156.4	-
TRIS	1	NaP1-TRIS-1	-	-	-	107.5	-

^a^ PA = palmitic acid; OA = oleic acid; LA = lauric acid; DAP = diaminopropane and EDA = ethylenediamine; TMEDA = *N*,*N*,*N*′,*N*′-tetramethylethylenediamine; TRIS = Tris(2-aminoethyl)amine. ^b^ BET SSA: specific surface area as measured using BET method. ^c^ CRC: CO_2_ retention capacity is expressed in micromoles of desorbed CO_2_ per gram adsorbent. ^d^ WRC: water retention capacity is expressed in micromoles of desorbed water per gram adsorbent. ^e^ Raw NaP1: as-synthesized and unwashed NaP1; NaP1: NaP1 washed. ^f^ CRC measured by TPD performed at 5 °C.min^−1^ heating rate from 20 °C to 450 °C under a 5 mL.min^−1^ nitrogen stream; ^g^ CRC measured by a similar TPD method but ranging from 20 °C to 120 °C, followed by stepwise thermal desorption (STD) at a constant temperature of 120 °C for 20 min.

## Data Availability

The data generated during this study can be obtained upon request to the corresponding authors.

## Supplementary Materials

The following supporting information can be downloaded at: https://www.mdpi.com/article/10.3390/molecules29040768/s1. Figure S1. Thermogravimetry patterns of NaP1 (in the range 100–75%) and of fatty acid loaded NaP1 (NaP1-FA-5, (in the range100–95.5%) %). Figure S2. FTIR spectra of NaP1 and of its 10% diaminopropane-loaded counterpart (NaP1-DAP-10). Figure S3. FTIR spectra of NaP1 before and after incorporation of similar 1% contents of different amines and different amounts of palmitic acid. Figure S4. FTIR spectra of different zeolites after incorporation of similar 1% diaminopropane contents. Figure S5. FTIR spectra of different zeolites after incorporation of similar 1% TRIS contents. Figure S6. Rise of the 2425 cm^−1^ band after exposure to air of NaP1 previously stored in a dry CO_2_-free enclosure for several days. Figure S7. XRD patterns of NaP1 with (a) 1%, (b) 5%, (c) 10%, (d) 20, and (e) 30% of palmitic acid content. P: Zeolite NaP1; M: Mullite, a crystalline aluminosilicate. Figure S8. Energy dispersion X-ray fluorescence and two scanning electron micrographic images of NaP1-PA-10. Figure S9. CO_2_ TPD profile between 20 and 120 °C with stepwise thermal desorption (STD) at constant temperature of 120 °C for 20 min. These patterns correspond to combined TPD-STD diagrams of zeolites 4A, X, NaP1 and Y loaded with 1% of different amines. Figure S10. CO_2_ TPD profile between 20 and 120 °C and stepwise thermal desorption (STD) at constant temperature of 120 °C for 20 min. These patterns correspond to combined TPD-STD diagrams of NaP1 loaded with different fatty acids: palmitic acid, lauric acid and oleic acid in a concentration of 10%. The scale was enhanced to NaP1 10% lauric acid and 10% oleic acid in order to visualize the peaks, the comparison is shown at Figure S10. Table S1. Chemical composition of NaP1 and FA-loaded counterparts as assessed by EDS. Table S2. CO_2_ Retention Capacity (CRC) for some zeolites modified with 1 wt.% of different amines.
