# Supplementary material for: Surface Basicity and Hydrophilic Character of Coal Ash-Derived Zeolite NaP1 Modified by Fatty Acids
Source: Molecules. 2024 Feb 7;29(4):768. doi: 10.3390/molecules29040768 (PMC10891618; doi:10.3390/molecules29040768)
Supplement: Supplementary file 1 [file molecules-29-00768-s001.zip › molecules-2838607-supplementary.pdf]

Surface Basicity and Hydrophilic Character of Coal Ash-Derived Zeolite NaP1 Modified by Fatty Acids

Ana-Paola Beltrão-Nunes [1,2], Marçal Pires [2,*], René Roy [1] and Abdelkrim Azzouz [1,3,*]

[1] Nanoqam, Department of Chemistry, University of Quebec at Montreal, Montreal, QC H3C 3P8, Canada; anapaolabn@gmail.com (A.-P.B.-N.); roy.rene@uqam.ca (R.R.)
[2] Graduation Program on Engineering and Technology of Materials, School of Technology, Pontifical Catholic University of Rio Grande do Sul, Porto Alegre 90619-900, Brazil
[3] Station Expérimentale des Procédés Pilotes en Environnement, École de Technologie Supérieure, Montreal, QC H3C 1K3, Canada
* Correspondence: mpires@pucrs.br (M.P.); azzouz.a@uqam.ca (A.A.); Tel.: +1-514-987-3000 (ext. 4119) (A.A.); Fax: +1-514-987-4054 (A.A.)

SUPPORTING INFORMATION

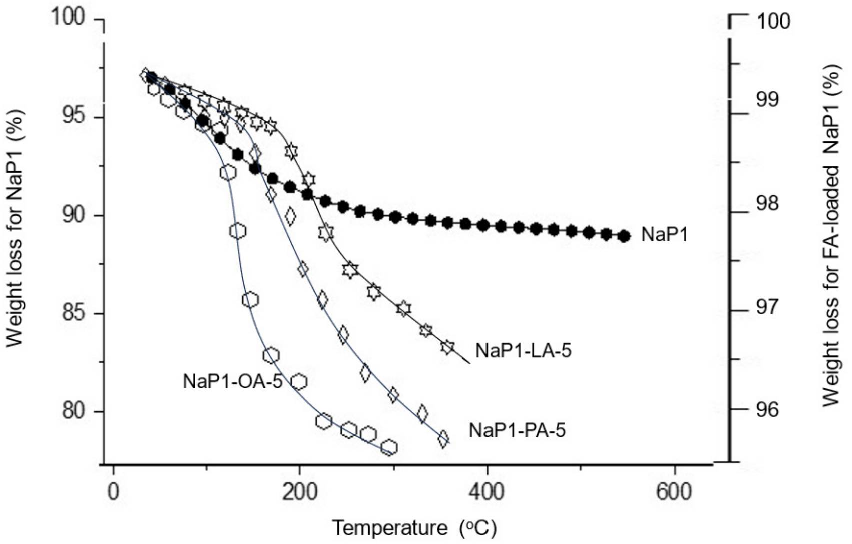

Figure S1. Thermogravimetry patterns of NaP1 (in the range100-75 %) and of fatty acid loaded NaP1 (NaP1-FA-5, (in the range100-95.5 %) %).

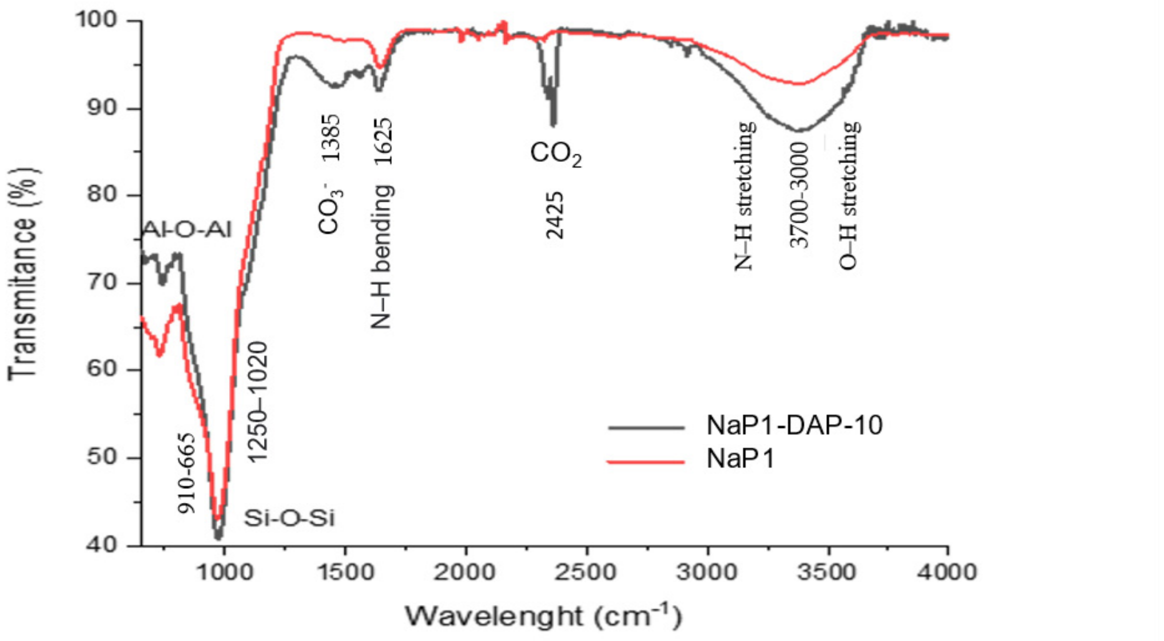

Figure S2. FTIR spectra of NaP1 and of its 10 % diaminopropane-loaded counterpart (NaP1-DAP-10).

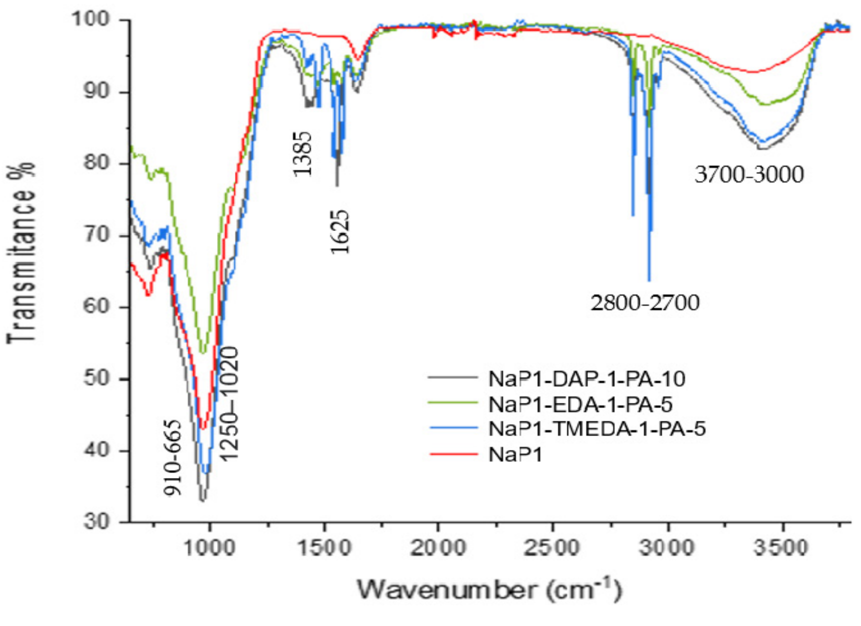

Figure S3. FTIR spectra of NaP1 before and after incorporation of similar 1 % contents of different amines and different amounts of palmitic acid.

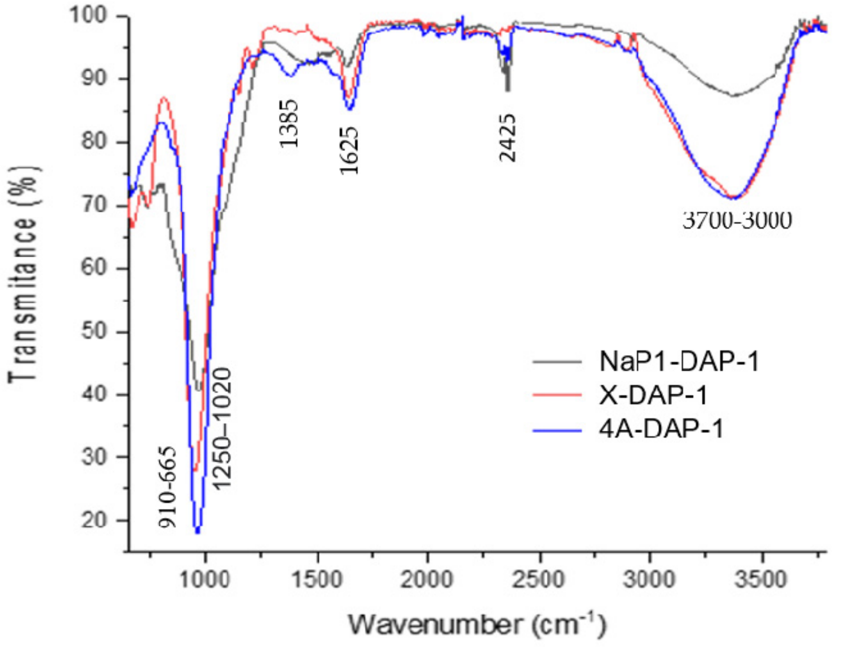

Figure S4. FTIR spectra of different zeolites after incorporation of similar 1 % diaminopropane contents.

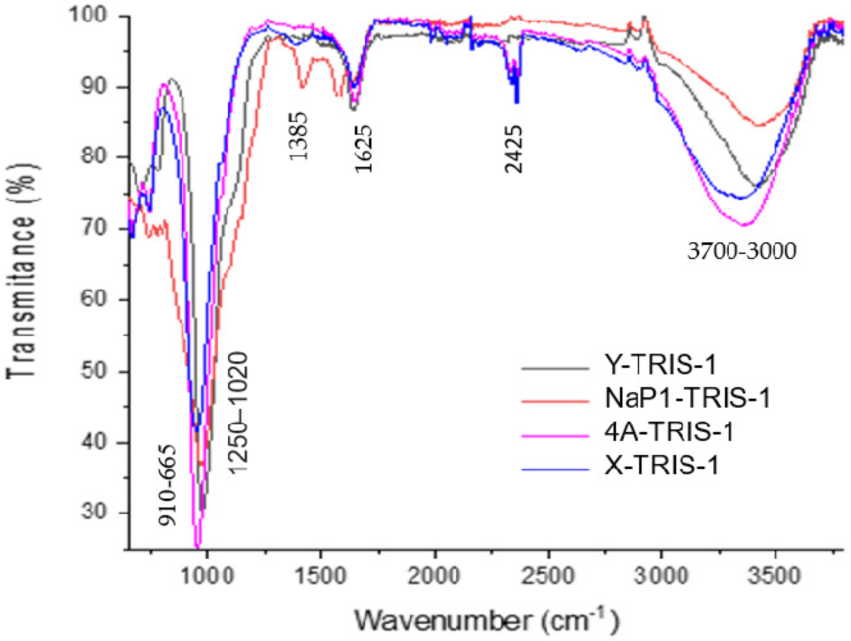

Figure S5. FTIR spectra of different zeolites after incorporation of similar 1 % TRIS contents.

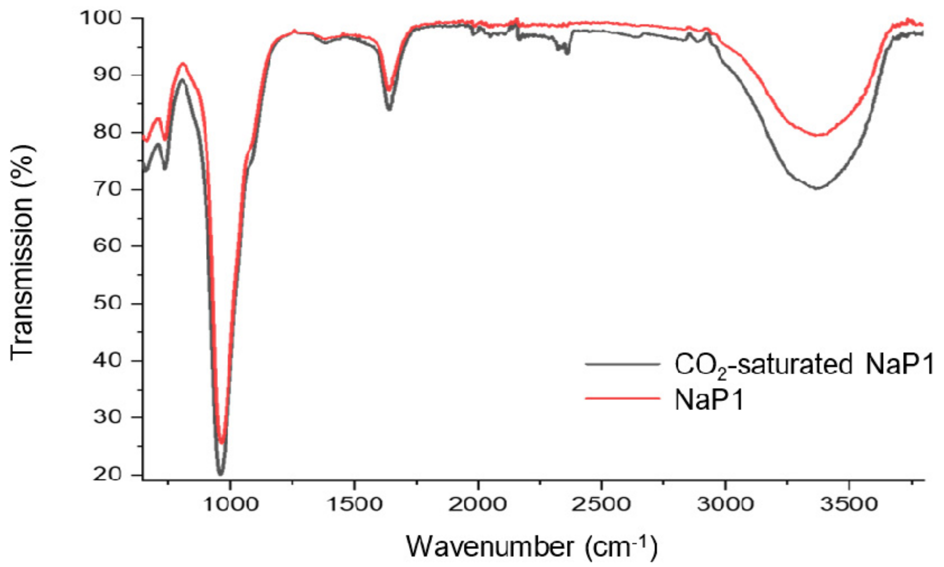

Figure S6. Rise of the 2425 cm^{-1} band after exposure to air of NaP1 previously stored in a dry CO_2-free enclosure for several days.

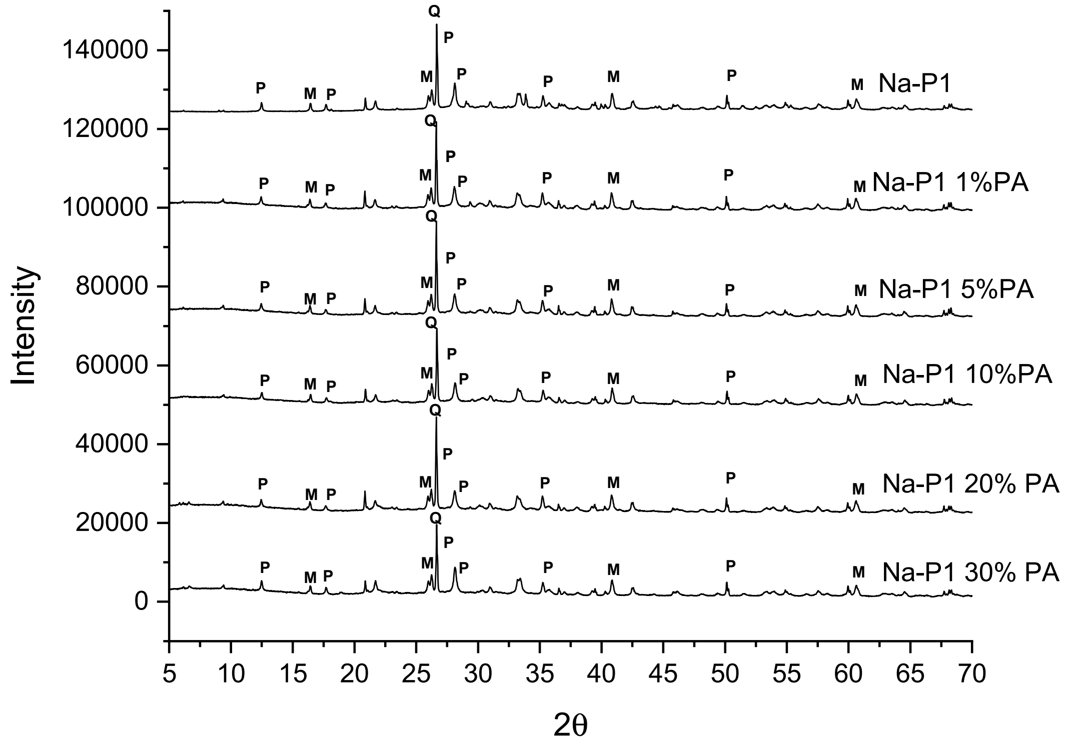

Figure S7. XRD patterns of NaP1 with (a) 1%, (b) 5%, (c) 10%, (d) 20, and (e) 30% of palmitic acid content.

P: Zeolite NaP1; M: Mullite, a crystalline aluminosilicate.

Table S1. Chemical composition of NaP1 and FA-loaded counterparts as assessed by EDS

	NaP1*	Palmitic acid content (%)			Lauric acid content (%)			Oleic acid content (%)		
Elements	(%)	1	5	10	1	5	10	1	5	10
Al	18.33	18.1467	17.23937	15.51543	18.1467	17.23937	15.51543	18.1467	17.23937	15.51543
C	0	0.75	4.4625	11.51625	0.72	4.284	11.0556	0.77	4.5815	11.82335
Ca	1.14	1.1286	1.07217	0.964953	1.1286	1.07217	0.964953	1.1286	1.07217	0.964953
Fe	3.78	3.7422	3.55509	3.199581	3.7422	3.55509	3.199581	3.7422	3.55509	3.199581
K	1.04	1.0296	0.97812	0.880308	1.0296	0.97812	0.880308	1.0296	0.97812	0.880308
Mg	0.57	0.5643	0.536085	0.482477	0.5643	0.536085	0.482477	0.5643	0.536085	0.482477
Na	7.21	7.1379	6.781005	6.102905	7.1379	6.781005	6.102905	7.1379	6.781005	6.102905
Ni	0.12	0.1188	0.11286	0.101574	0.1188	0.11286	0.101574	0.1188	0.11286	0.101574
O	45.55	45.2145	43.55378	40.3984	45.2545	43.79178	41.0126	45.2045	43.49428	40.24485
S	0.241	0.23859	0.226661	0.203994	0.23859	0.226661	0.203994	0.23859	0.226661	0.203994
Si	20.81	20.6019	19.57181	17.61462	20.6019	19.57181	17.61462	20.6019	19.57181	17.61462
Ti	0.8	0.792	0.7524	0.67716	0.792	0.7524	0.67716	0.792	0.7524	0.67716
Other traces	0.5	0.495	0.47025	0.423225	0.495	0.47025	0.423225	0.495	0.47025	0.423225
Total	100.091	99.96009	99.31209	98.08088	99.97009	99.37159	98.23443	99.97009	99.37159	98.23443

* The chemical composition of NaP1 as assessed by EDS-based XR-fluorescence underwent no change upon FA incorporation, and consequently was taken as the reference for the determination of those of FA-loaded NaP1 samples. The total percent is not equal to 100 %, because some light elements such hydrogen, helium, lithium and berylium are not detected by this technique.

Table S2. CO_2 Retention Capacity (CRC) for some zeolites modified with 1 wt.% of different amines.

Zeolites	CO_2 Retention Capacity (CRC) (μmol.g^{-1})					
	Amine	None	DAP	TMEDA	TRIS	EDA
X		285.7	53.7	33.7	81.1	52.5
NaP1*		296.1	289.9	156.4	107.5	220.8
Raw NaP1**		399.2	-	-	-	-
Y		78.1	94.6	28.5	22.5	41.0
4A		170.4	86.9	17.9	46.9	166.1

*NaP1: NaP1 washed; ** Raw NaP1: as-synthesized and unwashed NaP1.

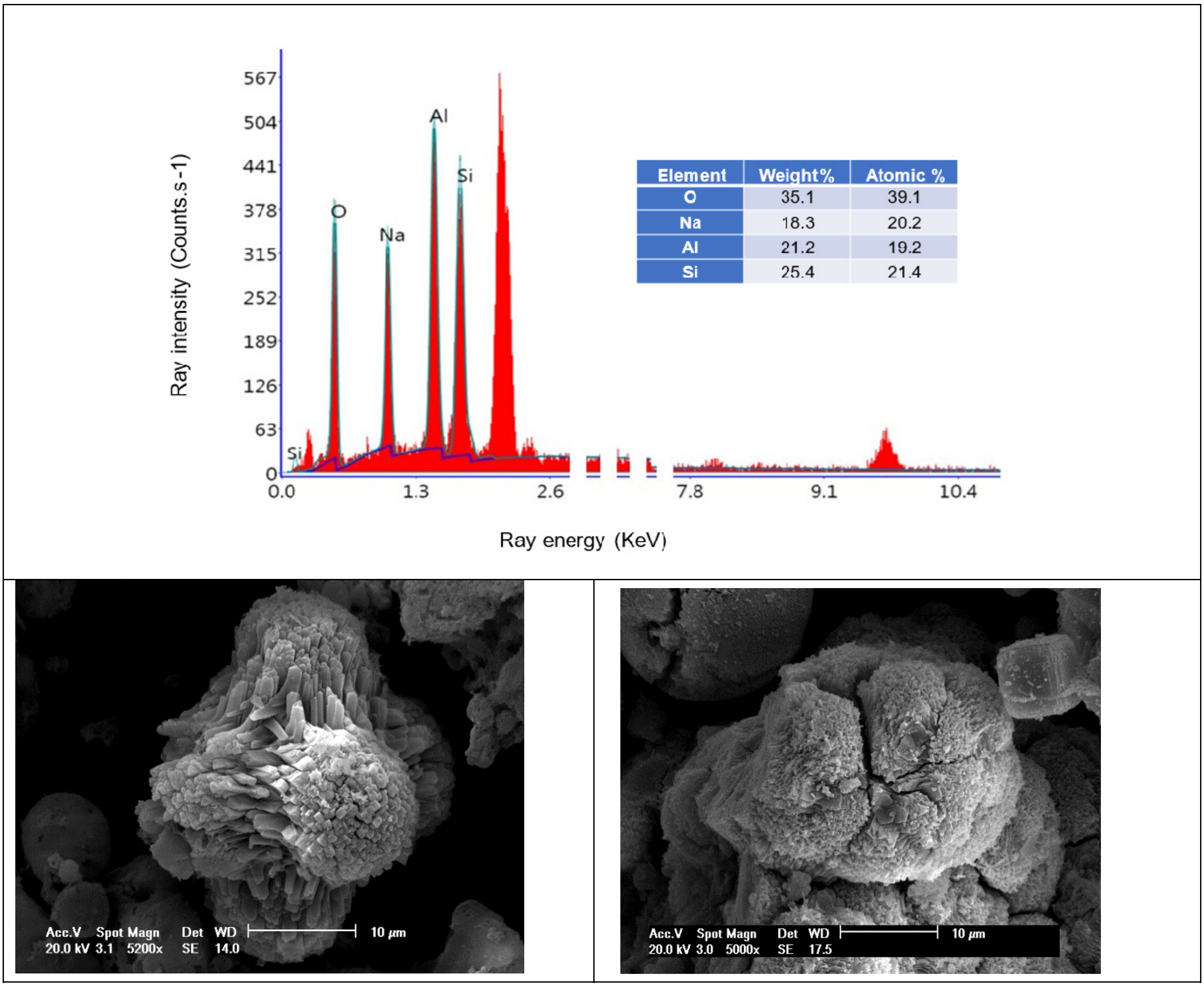

Figure S8. Energy dispersion X-ray fluorescence and two scanning electron micrographic images of NaP1-PA-10.

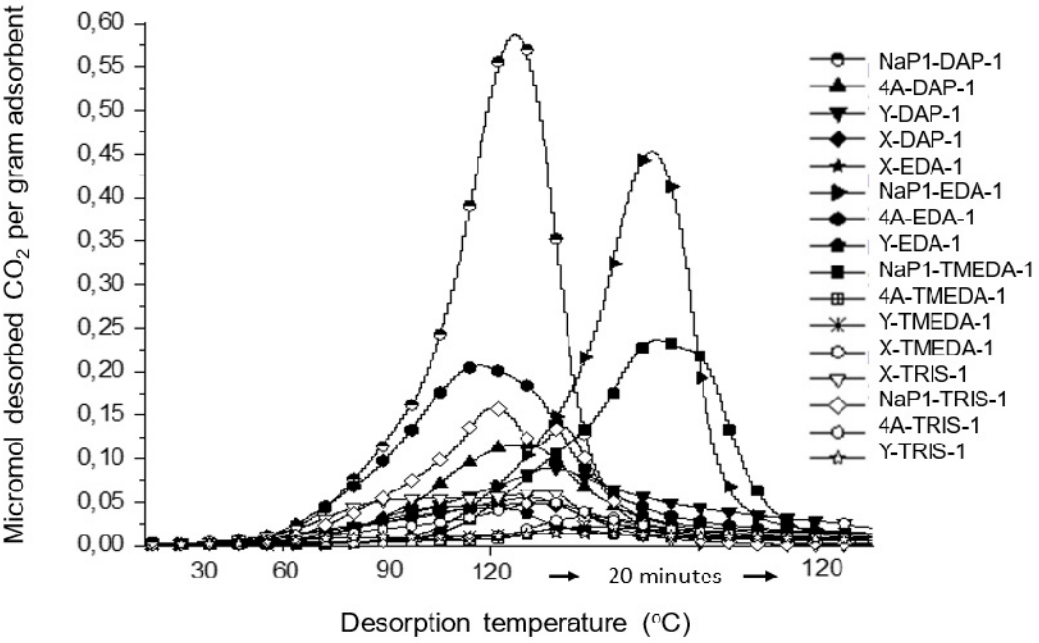

Figure S9. CO_2 TPD profile between 20 and 120 °C with stepwise thermal desorption (STD) at constant temperature of 120 °C for 20 min. These patterns correspond to combined TPD-STD diagrams of zeolites 4A, X, NaP1 and Y loaded with 1 % amount of different amines.

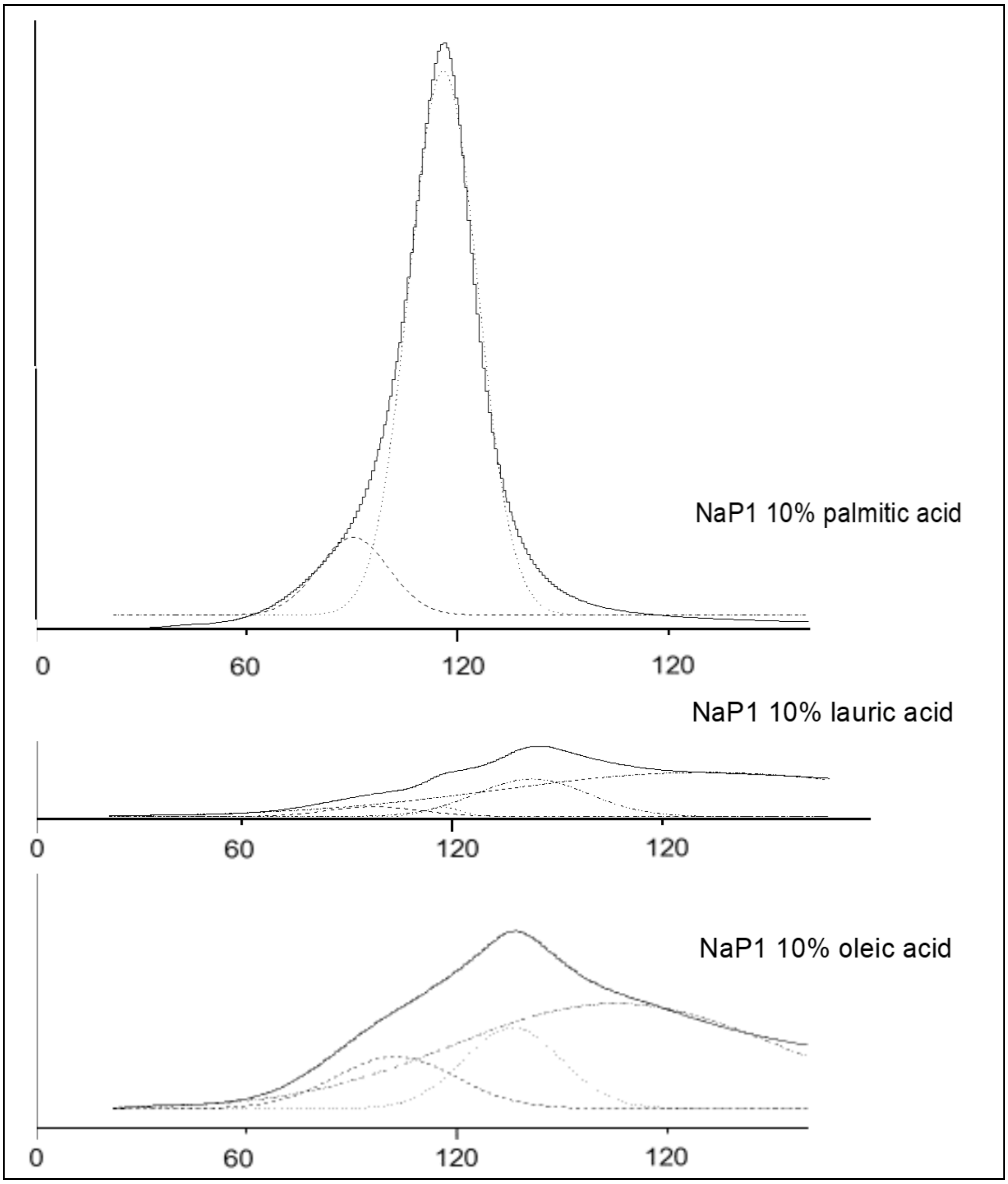

Figure S10. CO_2 TPD profile between 20 and 120 ºC and stepwise thermal desorption (STD) at constant temperature of 120 ºC for 20 min. These patterns correspond to combined TPD-STD diagrams of NaP1 loaded with different fatty acids: palmitic acid, lauric acid and oleic acid in a concentration of 10%. The scale was enhanced to NaP1 10% lauric acid and 10% oleic acid in order to visualize the peaks, the comparison is shown at Fig.S10.
